# Recent Advances in Kaolinite Nanoclay as Drug Carrier for Bioapplications: A Review

**DOI:** 10.1002/advs.202300672

**Published:** 2023-06-21

**Authors:** Qianwen Wu, Juan Liao, Huaming Yang

**Affiliations:** ^1^ Hunan Key Laboratory of Mineral Materials and Application School of Minerals Processing and Bioengineering Central South University Changsha 410083 China; ^2^ Engineering Research Center of Nano‐Geomaterials of Ministry of Education China University of Geosciences Wuhan 430074 China; ^3^ Laboratory of Advanced Mineral Materials China University of Geosciences Wuhan 430074 China; ^4^ Faculty of Materials Science and Chemistry China University of Geosciences Wuhan 430074 China

**Keywords:** bioapplication, drug carrier, kaolinite nanoclay, physicochemical properties, structure

## Abstract

Advanced functional two‐dimensional (2D) nanomaterials offer unique advantages in drug delivery systems for disease treatment. Kaolinite (Kaol), a nanoclay mineral, is a natural 2D nanomaterial because of its layered silicate structure with nanoscale layer spacing. Recently, Kaol nanoclay is used as a carrier for controlled drug release and improved drug dissolution owing to its advantageous properties such as surface charge, strong biocompatibility, and naturally layered structure, making it an essential development direction for nanoclay‐based drug carriers. This review outlines the main physicochemical characteristics of Kaol and the modification methods used for its application in biomedicine. The safety and biocompatibility of Kaol are addressed, and details of the application of Kaol as a drug delivery nanomaterial in antibacterial, anti‐inflammatory, and anticancer treatment are discussed. Furthermore, the challenges and prospects of Kaol‐based drug delivery nanomaterials in biomedicine are discussed. This review recommends directions for the further development of Kaol nanocarriers by improving their physicochemical properties and expanding the bioapplication range of Kaol.

## Graphical abstract

This review summarizes the recent progress of Kaol nanoclay for therapeutic delivery. Their naturally layered structure, abundant surface charge as well as strong biocompatibility, make Kaol a superior 2D nanocarrier. By summarizing the modification strategies and addressing the bio‐safety concerns, Kaol‐based nanocarriers were applied for antibacterial, anti‐inflammatory, and antitumor therapy.

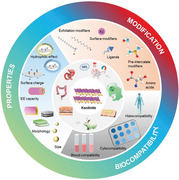

## 1 Introduction

The primary drawbacks of traditional drug delivery techniques include low solubility, rapid clearance, and side effects. As a controllable nanotechnology platform, nanocarriers can provide distinct benefits over conventional medication delivery techniques.^[^ ^1^, ^2^ ^]^ These nanostructures are employed as carriers or transport agents for vaccines,^[^ ^3^ ^]^ drugs,^[^ ^4^ ^]^ deoxyribonucleic acids (DNAs),^[^ ^5^ ^]^ proteins,^[^ ^6^ ^]^ and other substances required for the treatment of several diseases. Owing to their properties, nanocarriers can achieve various therapeutic outcomes in the treatment of various diseases. For example, they are widely used for diagnostics in neuroscience because of their size and ability to facilitate drug delivery to the blood–brain barrier.^[^ ^7^, ^8^ ^]^ Different nanomaterials such as nanoparticles (NPs), nano emulsions, solid lipid NPs, and liposomes have been used to treat neurodegenerative diseases.^[^ ^9^, ^10^, ^11^ ^]^ Because nanocarriers exhibit a spontaneous tendency toward phagocytosis and susceptibility to isolation by innate immune cells, they are particularly beneficial for targeting immune diseases involving the contribution of circulating immune complexes and local macrophages,^[^ ^12^ ^]^ dendritic cells,^[^ ^13^ ^]^ and neutrophils,^[^ ^14^ ^]^ which can be used as immunotherapeutic platforms for inflammatory and autoimmune diseases.^[^ ^15^, ^16^ ^]^ Moreover, they exhibit properties of enhanced drug permeability and prolonged drug release.^[^ ^17^ ^]^ Especially, the treatment of ocular diseases with low ocular bioavailability caused by insufficient retention time and reduced corneal epithelial permeability due to tear secretion.^[^ ^18^ ^]^ Nanocarriers are currently divided into three categories based on their composition: biological, organic, and inorganic nanomaterials.^[^ ^5^, ^19^ ^]^

Biological nanocarriers include three major categories based on their primary composition of proteins (gelatin, albumin, and silk protein),^[^ ^20^ ^]^ polysaccharides (chitosan, sodium alginate, cyclodextrin, and pectin),^[^ ^21^ ^]^ or nucleic acids.^[^ ^5^, ^22^ ^]^ Several physiological phenomena and diseases of the human body occur at the nanometric scale; among the nanoscale molecular machines, proteins as the bearers of life activities and nucleic acids as the carriers of genetic information bear most of the physiological activities in the organism.^[^ ^20^ ^]^ The unique characteristics of biological nanocarriers facilitate the development of ideal natural drug carrier materials.^[^ ^21^ ^]^ Regrettably, the research on biological nanomaterials is still in study.

Organic nanocarriers, including liposomes,^[^ ^23^ ^]^ dendrimers,^[^ ^24^ ^]^ polymeric nanocarriers,^[^ ^25^ ^]^ micelles,^[^ ^26^ ^]^ and viral nanocarriers, are versatile, less toxic, and capable of binding a variety of pharmaceuticals as well as drug delivery ligands.^[^ ^27^, ^28^, ^29^ ^]^ Among these, liposomes were previously studied as drug carriers, and a lot of research experience and clinical trial data have been accumulated; many related products have been marketed, such as Vyxeos,^[^ ^30^ ^]^ a liposomal formulation of erythromycin/alglucoside for the treatment of leukemia, and Arikayce,^[^ ^31^ ^]^ a liposomal suspension of amikacin sulfate for the treatment of lung diseases.

Inorganic nanocarriers include carbon nanomaterials,^[^ ^32^ ^]^ silica NPs, calcium nanomaterials,^[^ ^33^ ^]^ gold NPs,^[^ ^34^ ^]^ magnetic NPs,^[^ ^35^ ^]^ nano‐clay,^[^ ^36^ ^]^ and quantum dots.^[^ ^37^ ^]^ Inorganic nanocarriers are easy to handle and can be effectively used for biosensing, cell labeling, targeting, imaging, and diagnostics.^[^ ^38^, ^39^ ^]^ Relevant products based on inorganic nanomaterials have already entered clinical trials, such as the gold silica nano drug Auro‐Lase with polyethylene glycol (PEG) coating for infrared‐triggered thermal ablation therapy of solid tumors.^[^ ^39^, ^40^ ^]^ Inorganic nanocarriers can exhibit a stimulus‐responsive release function that can specifically release drugs in response to external triggers (heat, light, and magnetic fields) in a tumor microenvironment to avoid recognition of anticancer drugs by drug efflux transport proteins;^[^ ^41^, ^42^ ^]^ at the same time, they can carry therapeutic genes for synergistic therapeutic effects because of their easily modified surfaces.^[^ ^22^ ^]^ Moreover, they can provide a multifunctional platform for cancer therapy, such as thermotherapy.^[^ ^43^ ^]^ More importantly, these inorganic nanocarriers can also enable molecular imaging, which aids in monitoring the drug delivery process and treatment outcomes to improve therapeutic efficacy.^[^ ^44^ ^]^ Inorganic materials are promising candidates for drug delivery systems (DDSs).

Therefore, researchers are still working on new materials and strategies to design safe, therapeutically effective, and patient‐compatible nanocarriers for DDSs. Environmentally friendly clays, the layered mineral silicates,^[^ ^45^ ^]^ have been performing an essential role in anti‐diarrhea treatment,^[^ ^46^ ^]^ as hemostatic agents,^[^ ^47^ ^]^ for gastrointestinal protection,^[^ ^48^ ^]^ as antibacterial antiviral agents, and in other biomedical sectors. This class of materials has got attention over other nanocarriers owing to their rich mineral resources and unique properties of dissolution, intercalation, structure, and adsorption.^[^ ^49^, ^50^, ^51^ ^]^ Kaolinite (Kaol) is the most produced clay mineral worldwide with high biocompatibility,^[^ ^52^ ^]^ biostability, and adsorption properties,^[^ ^53^ ^]^ and is widely used in skin inflammation,^[^ ^53^ ^]^ antibacterial,^[^ ^54^, ^55^ ^]^ hemostasis,^[^ ^52^ ^]^ and wound healing applications.^[^ ^53^ ^]^ Moreover, the significantly active hydroxyl group between the layers serves as active sites for Kaol modification, making Kaol a superior two–dimensional(2D) nanocarrier for the efficient delivery of drugs by modifying the release (rate or time),^[^ ^52^, ^56^ ^]^ increasing the stability of the drug, improving the dissolution profile of a drug, or enhancing its intestinal permeability. It can significantly enhance the therapeutic effect on malignant tumors and resolve the issues of limited water solubility, poor selectivity, and harmful side effects of therapeutic molecules. Various drugs, ranging from anticancer drugs^[^ ^57^, ^58^ ^]^ to non‐cancer drugs^[^ ^56^, ^59^ ^]^ and organic^[^ ^60^ ^]^ and therapeutic molecules have been successfully loaded onto Kaol‐based nanocarriers and have exhibited a sustained‐release profile. Although the presence of intercalating guest molecules in the Kaol interlayer space is problematic because of the properties of low expansion and low cation‐exchange capacity, modification methods have introduced a way to develop a variety of nanohybrid carriers based on Kaol by designing and selecting appropriate and desirable nanocarriers with functional groups or linkers, which can improve drug delivery and promote drug release at appropriate times and sites. Another challenge is the development and fabrication of simple drug carriers that respond to physiological microenvironments for optimized therapeutic benefits. Understanding how Kaol is used in drug delivery is essential for further investigation of its biofunctionalization. In this review, we describe a Kaol‐based carrier with controlled and sustained‐release properties for therapeutic delivery.

## 2 Size, Morphology, and Properties of Kaol

**Figure 1:**
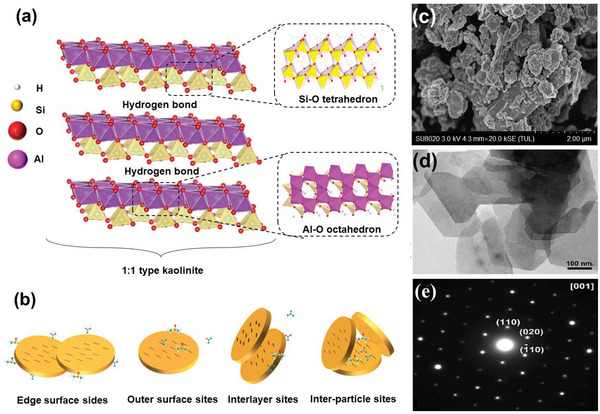
a) Structural model of Kaol crystal. b) Interaction sites of Kaol with functional molecules. Reproduced with permission.^[^ ^64^ ^]^ Copyright 2019, Elsevier. c) SEM image of Kaol sheets. Reproduced with permission.^[^ ^65^ ^]^ Copyright 2019, Elsevier. d) TEM image of Kaol sheets. e) Selected‐area electron diffraction of Kaol.d,e) Reproduced with permission.^[^ ^66^ ^]^ Copyright 2019, Elsevier.

Kaol is a layered silicate mineral with the chemical composition of Al_2_[Si_2_O_5_](OH)_4_, which can also be expressed as A1_2_O_3_·2SiO_2_·2H_2_O, where water exists in the form of hydroxyl groups.^[^ ^61^ ^]^ Theoretically, the percentage composition of A1_2_O_3_, SiO_2_, and H_2_O in Kaol are 41.2%, 48.0%, and 10.8%, respectively. Kaol consists of Si—O tetrahedra and A1—O octahedra that are combined in a 1:1‐type structure,^[^ ^62^ ^]^ where the Si—O tetrahedra and A1—O octahedra share oxygen atoms through hydrogen bonds between aluminum (Al—OH) and silanol (Si—O) groups.^[^ ^63^ ^]^ The structural model is shown in **Figure** **1**a. The particle size of the pseudohexagonal pure Kaol platelets is typically <2 µm;^[^ ^49^ ^]^ the shape and size are illustrated in Figure 1c–e, which were captured using scanning electron microscopy (SEM), transmission electron microscopy (TEM), and selected‐area electron diffraction, respectively. Kaol belongs to the triclinic crystal system,^[^ ^67^ ^]^ in which the cell parameters are *a*_0_ = 0.514 nm, *b*_0_ = 0.893 nm, *c*_0_ = 0.737 nm, *α* = 91.8°, *β* = 104.30°, and *γ* = 90°. The (001), (010), and (110) surfaces are the common cleavage planes of Kaol crystals,^[^ ^68^ ^]^ where the (001) plane is the basal plane and the (010) and (110) planes are the end planes.^[^ ^69^ ^]^ When cleavage of Kaol occurs along the (001) plane, only interlayer hydrogen bond breakage occurs^[^ ^70^ ^]^ instead of chemical bond breakage; consequently, theoretically, the surface is electrically neutral.^[^ ^71^ ^]^ However, in the natural environment, Al^3+^ is replaced by Ca^2+^, Mg^2+^, or Fe^2+^, and Si^4+^ is replaced by Al^3+^ or Fe^3+^, resulting in an insufficient charge in the crystal lattice and a negatively charged surface. Notably, the (110) and (010) surfaces exhibit pH‐dependent amphoteric charges caused by the protonation and deprotonation of hydroxyl groups,^[^ ^72^ ^]^ which are positive at low pH and turn negative with an increase in pH. The charged characteristics of Kaol result in the sequestration of functional molecules at four types of action sites: edge surface (end surface), outer surface, interlayer, and interparticle sites,^[^ ^73^ ^]^ suggesting that Kaol can function as a pH‐responsive drug nanocarrier (Figure 1b).

### 2.1 Size and Morphology of Kaol‐Based Carrier

**Figure 2:**
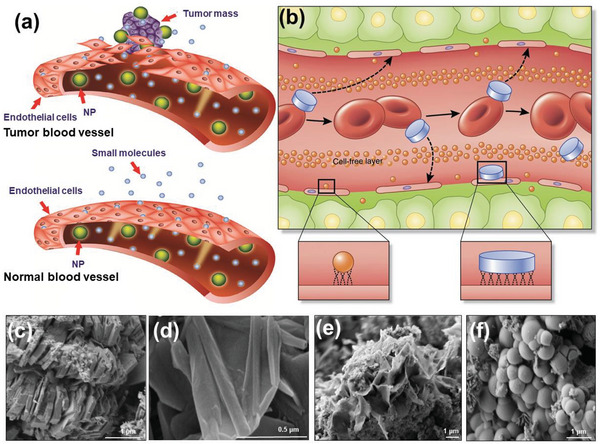
a) Tumor blood vessels have large openings between endothelial cells (ECs), allowing NPs to reach the stroma and tumor cells. In contrast, normal tissues contain tightly connected ECs that prevent the diffusion of NPs from blood vessels. a) Reproduced with permission.^[^ ^77^ ^]^ Copyright 2015, Dove Medical Press Ltd). b) Longitudinal cross‐sectional illustration of a blood vessel containing circulating red blood cells (red, oval shapes), spherical particles (orange), and discoidal particles (blue, rectangle shapes) that travel through and adhere to the walls. Margination inset (right): lateral drifting of discoidal particles promotes periodic interaction with the vessel walls; margination inset (left): limited size and surface area of conventional spherical NPs contacting with ECs. Reproduced with permission.^[^ ^78^ ^]^ Copyright 2015, Springer Nature. c) The common booklet‐like shape of Kaol. d) The tubular morphology of Kaol treated by chemical modification. c,d) Reproduced with permission.^[^ ^79^ ^]^ Copyright 2015, Elsevier. Synthesis results of e) sponge‐shaped and f) spherical Kaol. Reproduced with permission.^[^ ^80^ ^]^ Copyright 2021, American Chemical Society.

The radial size and morphology of drug carrier particles that are used as biocompatible composites affect their interactions with the physiological environment during transportation. The optimal nanoparticle size should be between 10 and 200 nm,^[^ ^74^ ^]^ where particles of 25 nm diameter can diffuse over longer distances and times, and particles with diameters close to 100 nm exhibit superior body circulation and tumor accumulation.^[^ ^75^ ^]^ Moreover, 1000 diameter nm × 400 height nm discoidal NPs are more likely to produce specific adhesions in tumor capillary walls through vascular adhesion mechanisms, resulting in quicker tumor transport than that ensuing from the enhanced permeability and retention effect.^[^ ^75^, ^76^ ^]^ In the study of tumor‐targeted nanocarriers, the vascular wall gap in normal tissues is generally below 5 nm, while the size range of the vascular gap in tumor tissues is ≈400–800 nm, which implies that large‐sized NPs are more likely to be enriched at tumors and less likely to cross normal tissues (**Figure** **2**a). Extensive research has been conducted to determine the optimal delivery size of Kaol with different morphologies. The diameter of Kaol in a roll‐shaped structure is ≈20 nm, whereas the diameter of Kaol in a sheet‐shaped structure is approximately in the range of 150 to 1000 nm. Although the optimal size of the nanoclay carrier is 200 nm,^[^ ^36^ ^]^ studies demonstrated that Kaol‐based DDSs between 200 and 400 nm could be internalized into cytoplasmic lysosomes but failed to cross the nuclear membrane.^[^ ^81^ ^]^ Moreover, a Kaol carrier with a size ranging between 400 and 500 nm showed pH‐responsive behavior by permeating the blood barrier.^[^ ^36^ ^]^ However, the optimal delivery size range of Kaol in the DDSs is still unclear. Future studies on the size design need to maintain drug loading and delivery efficiency, while also considering the impact of its in vivo biological distribution, cell uptake methods, and clearance pathways, and finalizing an optimal delivery size range.

The nanoscale morphological features also play an essential role in the physical and chemical properties of Kaol as a carrier.^[^ ^82^ ^]^ The morphological differences result in different in vivo behaviors, which affect the movement mode of the infiltration process in the systemic circulation and tumor microenvironment, thereby affecting the drug‐loading capacity and cytotoxicity. For example, discoidal particles are more likely to be marginated into the vessel wall to establish contact/binding sites with ECs than conventional spherical NPs because of the ability of non‐spherical particles to exhibit flip‐and‐roll kinetics in a vessel (Figure 2b).^[^ ^78^ ^]^ Moreover, the easier uptake of tubular nanomaterials by cells when compared to that of spherical NPs is attributed to the fact that they may enter cells directly through the cell membrane in addition to the endocytosis of nanomaterials by cells, resulting in increased cellular uptake and better therapeutic effects.^[^ ^75^ ^]^ Kaol usually exhibits sheet morphologies with a common booklet‐like shape (Figure 2c).^[^ ^49^ ^]^ It is often modified from its natural morphology by physical or chemical treatment to tubular morphology to enhance the material's delivery properties (Figure 2d).^[^ ^83^, ^84^ ^]^ Kaol nanotubes (KNTs) are good mesoporous materials with homogeneous inner cavities that provide reaction sites for chemical reactions such as ion transport and molecular sustained load and release. The diameter of KNTs normally ranges from 20 to 100 nm, and the length depends mainly on the diameter of the raw Kaol, which ranges from 250 to 2000 nm. Their morphological and property diversity makes them a valuable alternative to natural halloysite nanotubes (HNTs). This is due to the restorative forces caused by dislocations in the tetrahedral and octahedral structures of Kaol unit layers drive the auto‐curling of individual flakes or sheets. The curl exposes more active sites and is readily bound to functional molecules. Li et al.^[^ ^85^ ^]^ concluded that KNTs have a higher specific surface area and different types of pore spaces than natural HNTs, resulting in a higher pore‐space ratio. The proportion of pore space was 11% for HNTs and 50% for KNTs, resulting in a significant increase in the loading space where guest molecules such as the anticancer drug 5‐fluorouracil (5‐FU) can be infused.^[^ ^86^ ^]^ Moreover, dried gels corresponding to the chemical formula of Kaol were used to synthesize sponge‐shaped and spherical Kaol (Figure 2e,f), and the relationship between the cytotoxicity of these aluminum silicate NPs and their morphologies was investigated by initiating contact with human histiocytic lymphoma cells.^[^ ^80^ ^]^ The half‐maximal inhibitory concentrations were 1.55, 2.68, and 4.69 mg mL^−1^ for the samples with flat, spherical, and nano‐sponge morphologies, respectively, demonstrating that the toxicity of NPs to tumor cells was morphology dependent and the flat‐shaped NPs were the most toxic to tumor cells. The above studies proved that particle morphology significantly affects the drug‐loading capacity and cytotoxicity of Kaol‐based carriers.

### 2.2 Physicochemical Properties of Kaol‐Based Carriers

**Figure 3:**
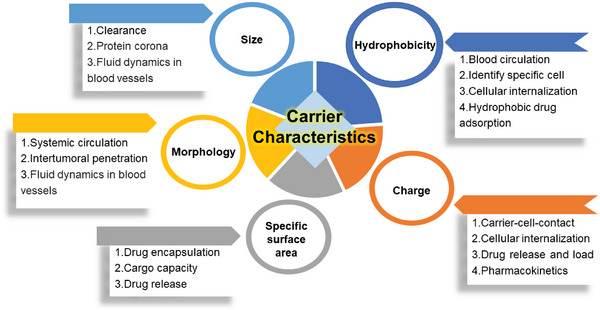
Influence of the carrier characteristics of Kaol.

The size and morphology are the essential characteristics affecting the biodistribution, destiny in vivo, and drug delivery efficiency of nanocarriers. Furthermore, the proper design of other physicochemical properties also allows the nanocarriers to reach tumor sites effectively and deliver drugs uniformly in the tumor tissue. The effects of the physical and chemical properties of Kaol on drug carriers are shown in **Figure** **3**. First came the charge characteristics, which is the main force that drives nanocarriers close to the cell surface and induces cellular internalization.^[^ ^87^ ^]^ On the one hand, amphoteric or negatively charged nanocarriers facilitate prolonged circulation time in vivo. On the other hand, positively charged nanocarriers are easily internalized by cancer cells because of their negatively charged cellular membranes.^[^ ^76^ ^]^ Thus, the charge characteristics affect the pharmacokinetics and intratumoral transport of Kaol.^[^ ^88^ ^]^ Furthermore, the surface electricity of Kaol affects the degree of drug loading and release behavior. The surface charge of untreated Kaol is always negative in the pH range of 2.5 to 11.0 and increases with increasing pH,^[^ ^89^ ^]^ which indicates that Kaol carriers have the potential to widely load cationic drugs. Moreover, the variation in the edge charge reflects the ability of the Kaol‐based carriers to control release.^[^ ^81^ ^]^ In a study on the adsorption of doxorubicin (DOX) by dodecylamine (DDA)‐modified Kaol (Kaol_C12N_) composites, the cumulative release of DOX in pH 4.0, 5.5, and 7.4 buffer solutions (representing extracellular tumor conditions, endosomal–lysosomal conditions, and blood conditions, respectively) were observed to be 23.20%, 9.20%, and 2.96%, respectively, which was attributed to the C_12_N modification resulting in an extensive zeta potential profile for Kaol.

The hydrophilic effect of nanocarriers helps in prolonging blood circulation and identifying specific areas on the cell surface. Moreover, the switch to hydrophobicity is effective for tumor tissues, and certain hydrophobic drug molecules, such as DOX and paclitaxel, can be efficiently adsorbed onto hydrophobic surface carriers. Kaol is a hydrophilic mineral as the outer surface of pure Kaol contains µ‐hydroxyl and water cations,^[^ ^90^ ^]^ which can be designed as nanocarriers to alter drug solubility based on the highly hydrophilic property. Researchers demonstrated that the wettability of phosphatidylcholine (PC)‐Kaol can be controlled by temperature, which stabilized a Pickering lotion containing the insoluble drug curcumin.^[^ ^90^ ^]^ However, Kaol can also be transformed into a hydrophobic carrier by modification, and its blood circulation and cell uptake abilities can be improved by adding a PEGylation procedure after loading hydrophobic drugs.^[^ ^81^ ^]^

Drug encapsulation or cargo capacity primarily depends on the specific surface area (pore/cavity size) of the NPs, as a large relative surface area increases drug adherence and the size of the pores influences the rate of drug release. The pore size of pure Kaol is ≈7 nm, which is sufficient for ensuring the existence of NPs and DOX in the interlayer and pore structures.^[^ ^91^ ^]^ Moreover, modification increases the pore/cavity size of Kaol prior to drug encapsulation to obtain a greater drug‐loading capacity. The specific surface area of Kaol that was modified using ultrasound‐assisted cetyltrimethylammonium chloride (CTAC) exfoliation was increased from 9.98 to 100.7 m^2^ g^−1^, and the total pore volume was increased from 0.049 to 0.48 cm^3^ g^−1^.^[^ ^92^ ^]^ When compared to pure Kaol, the DOX adsorption capacity of cetyltrimethylammonium bromide (CTAB)‐modified Kaol increased from 45% to 60%,^[^ ^91^ ^]^ which was attributed to the increase in the specific surface area, which caused a more cracked surface and finer pores in Kaol. *Tert*‐butyldimethylchlorosilane (TBSCI) was used to modify Kaol in the presence of an imidazole alkaline catalyst.^[^ ^93^ ^]^ The silane coupling agent treatment of Kaol produced pore sizes in the micrometer range, which is close to those of biomolecules, leading to a threefold increase in the adsorption of bovine serum albumin (BSA) as compared to that by natural Kaol.

Drug encapsulation efficiency (EE) is critical for the delivery of insoluble, unstable, or toxic compounds. Improving the EE of a drug in a carrier can result in greater therapeutic efficacy and minimal adverse effects. The low cation‐exchange capacity and specific surface area of natural Kaol result in a low EE of the guest drug. Marginal cation exchange on the surface of Kaol is the primary reason for its uptake of chlorpheniramine,^[^ ^94^ ^]^ with an adsorption capacity of only 25 mmol kg^−1^. Moreover, the content of the curcumin‐encapsulated emulsion of modified Kaol was 90%, when compared to a mere 60% content in the pure Kaol emulsion, which showed that modifications helped Kaol to increase the stability of the encapsulated curcumin.^[^ ^90^ ^]^ Moreover, methoxy‐modified Kaol (Kaol_MeOH_) achieved the interlayer insertion of the anticancer drug 5‐FU and provided additional 5‐FU adsorption sites via methoxy groups on the outer surface, resulting in a 5‐FU loading of 55.4% in Kaol_MeOH_, which was 2.66 times higher than that in pure Kaol.^[^ ^58^ ^]^ The drug release from NPs is crucial during evaluations in the preclinical development of drug formulations.^[^ ^95^ ^]^ While a slow release rate can result in insufficient release in the tumor with low bioavailability and modest therapeutic efficacy, a rapid release of drugs can result in systemic exposure and low distribution in the tumor.^[^ ^96^, ^97^ ^]^ The Kaol drug release profiles show that a period of 20 h is required to release the drug completely; however, these may be further modified using intercalation, grafting, or exfoliation to achieve superior drug release profiles.

## 3 Regulation Strategies of Kaol

**Figure 4:**
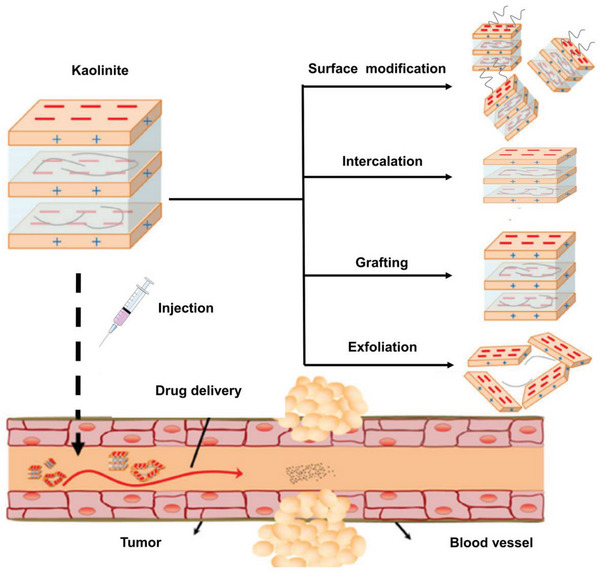
Schematic diagram of Kaol and the regulation strategies of Kaol used for tumor therapy. Reproduced with permission.^[^ ^89^ ^]^ Copyright 2017, Springer Nature.

Kaol is a biocompatible material that is used in biomedicine as a carrier of drug molecules. However, owing to the asymmetric distribution of atoms on both sides of the interlayer domain, the Kaol interlayer comprises polar molecules.^[^ ^98^ ^]^ The combined effect of interlayer hydrogen bonding and bipolarity results in solid bonds between Kaol sheets, making it difficult for compounds to be inserted into the Kaol interlayer. Therefore, the drug molecules are adsorbed on the end face of Kaol through weaker hydrogen bonds or van der Waals forces, leading to low loading and fast release.^[^ ^99^ ^]^ However, nanohybrids based on Kaol have the advantage of combining both the structural properties of Kaol and the chemical properties of the functional molecule as intercalated species. Researchers have proposed four modification strategies: surface modification, intercalation, grafting, and exfoliation, which are illustrated in **Figure** **4**. Surface modifiers are reacted with the hydroxide radicals on the surface of Kaol for changing the physicochemical properties of the Kaol surface to adapt it to the delivery environment for transporting functional molecules. Moreover, the rigid structure prevents Kaol from deforming during the interlayer reaction, which is helpful for the self‐assembly and molecular recognition of functional molecules between the layers. Increased interlayer spacing and adsorbed functional molecules create more space and binding sites for drug loading, thereby altering the physical and chemical characteristics of Kaol as a drug carrier (**Table** **1**).

**Table 1:** Summary of Kaol modification methods

Categories	Modifier	Molecular structure	Dipole moment [D]	Basal spacing [nm]	Intercalate ratio [%]	Action sites	Synthesis method	Refs.
Surface modification	TBSCI	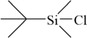	—	—	—	Formation of H—O—Si hydrogen bonds through Si with oxygen atoms of Si—OH tetrahedra	Atmospheric reaction	[93]
	3,4‐dihydroxybenzophenone	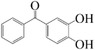	—	—	—	Formation with Al^3+^ of the aluminol basal surface	Solution	[100]
Intercalation	formamide	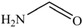	3.71	1.06	92.10	Si—OH and O—H on the inner surface form hydrogen bonds with NH and C=O, and NH was partially inserted into the Si—OH tetrahedra	Homogenization, solution	[101, 102]
	N–methylformamide (NMF)	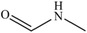	3.83	1.08	94.00	Formation of NH—O—Si hydrogen bonds through NH with oxygen atoms of Si—OH tetrahedra	Solution	[102, 103]
	hydrazine	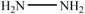	1.75	1.04	59.00	Four hydrogen atoms of hydrazine with Si—OH interlayer	Solution	[104]
	urea	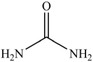	4.56	1.07	92.00	C=O and N—H form hydrogen bonds with the Al—OH interlayer, and the O of the Si—OH interlayer	Solution, aqueous suspension, homogenization	[102, 105]
	dimethyl sulfoxide (DMSO)	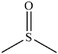	3.96	1.12	94.40	The C—S—C and CH_3_ are parallel to the Al interlayer, with one CH_3_ pointing to the center of the Si—OH tetrahedral	Solution, homogenization method	[106, 107]
Grafting modification	pyridine	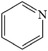	2.19	1.22	90.20	N form hydrogen bonds with hydroxyl groups on the Al—OH octahedron	—	[108]
	potassium acetate (Kac)	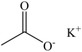	—	1.42	73.00	O on the carboxyl group form a hydrogen bond with the hydroxyl group of Al—OH octahedron	Solution	[109]
	methanol (MeOH)	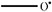	1.70	0.85	96.00	O—H with Al—O to form an Al—OH and H_2_O; an Al—O—C is formed by O—H and O of Al—OH interlayer	Solvothermal	[110]
	3,4‐dihydroxybenzophenone (APTES)	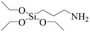	—	1.73–1.89	95.00	Electrostatic interactions between N—H and O of Al—OH interlayer	Solution/stirring, solvothermal	[111, 112, 113]
Exfoliation modification	ammonium salts	CTAB 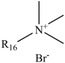	—	2.07	65.62	Si—O—Si groups interacted with CTAB^+^ cations	One‐step delamination	[114]
		CTAC 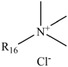	—	3.79	79.00	—	Solvothermal	[82]
		DDA 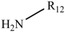	—	4.16	70.00	Interlayers in both paraffin‐type structures and distorted conformation Solution	Solution	[81]

### 3.1 Surface Modification

**Figure 5:**
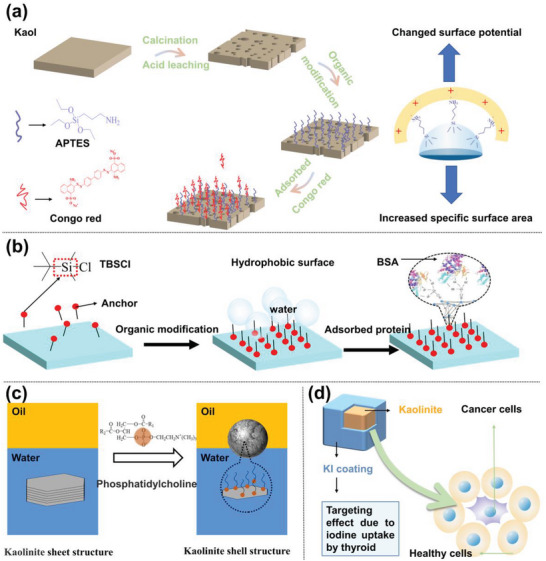
a) Organic modifier is reacted with the surface hydroxyl of Kaol to change the characteristics of the Kaol‐based carrier (Reproduced from^[^ ^115^ ^]^ with permission, copyright 2018, Elsevier); b) Illustration of surface modification of Kaol using a silane coupling agent. Reproduced with permission.^[^ ^93^ ^]^ Copyright 2017, Elsevier. c) PC contains a positively charged choline group and was easily adsorbed on the outer octahedral surface to change the hydrophobicity of Kaol; moreover, emulsification helped Kaol to form a dense shell structure to entrap drugs. Reproduced with permission.^[^ ^90^ ^]^ Copyright 2019, Elsevier). d) KI coated on Kaol surface to target thyroid tumors. Reproduced with permission.^[^ ^116^ ^]^ Copyright 2016, Springer Nature.

Owing to the structural characteristics of Kaol, the hydroxyl groups on the outer and edge surfaces are the major reaction sites on the Kaol surface; moreover, the primary method for modifying the Kaol surface is the surface reaction approach, which coats the Kaol surface with a layer of organic compounds by physical or chemical adsorption. The hydrolysis of silane coupling agents leads to the connection of active alcohol groups with —OH groups on the surface of Kaol, altering the physical and chemical properties of the Kaol‐based carrier surface and improving drug loading and release. After calcination, acid leaching, ultrasonic dispersion, and surface modification using APTES, the obtained Kaol nanosheets showed a significant increase in adsorption properties due to an increase in the specific surface area and pore structure of Kaol, as well as a change in the surface potential due to the protonation of amino groups, which exhibited superior adsorption of Congo red (**Figure** **5**a).^[^ ^115^ ^]^ In addition to the change in the Kaol adsorption capacity, the silane coupling agent can also change its hydrophilic and hydrophobic properties to improve the potential of Kaol for molecular loading by interacting with the biological environment. Selecting the surface grafting method of TBSCI and APTES is an effective way to change the surface property of Kaol. The transformation of hydrophilic to hydrophobic or electrophilic resulted in a 36% increase in the adsorption capacity for BSA (Figure 5b).^[^ ^93^ ^]^ Tang et al.^[^ ^90^ ^]^ used PC to modify Kaol, improving the wettability of the modified Kaol by modulating the modification temperature and increasing in contact angle from 96 to 139° during a temperature rise from 65 to 85 °C (Figure 5c). The improved wettability allowed the stable emulsion of modified Kaol to form a dense, ordered shell structure on the surface of the oil droplets, which successfully coated the drug to prevent erosion by simulated gastric juice. Drug‐loaded NPs are usually encapsulated in a responsive coating that controls the release of the carrier outside the cell to achieve drug delivery. Zhang et al.^[^ ^116^ ^]^ investigated the biodistribution of drug‐loaded Kaol coated with potassium iodide (Figure 5d) in the thyroid, liver, and kidney using inductively coupled plasma atomic emission spectroscopy; the results showed that the modified Kaol nanocarrier was accumulated significantly in the thyroid. At the same time, no significant increase was observed in accumulation in the liver and kidney, thus achieving the goal of targeting thyroid cancer cells via Kaol surface modification.

### 3.2 Intercalation Modification

The process by which organic matter is directly adsorbed or introduced into the interlayers of Kaol is called intercalation. Intercalation is a necessary precursor preparation step to obtain functional nanohybrids. Polar organic molecules that can be directly intercalated are classified into the following three categories:^[^ ^117^ ^]^ i) organic molecules with proton activity, such as formamide, NMF, hydrazine, and urea (U); ii) molecules with high dipole moments, such as DMSO and pyridine and its derivatives; iii) short‐chain fatty acid cations of potassium, rubidium, cesium, and ammonium such as Kac and potassium propionate. These polar molecules disrupt the interlayer hydrogen bonds of Kaol and create new hydrogen bonds to maintain the stability of the molecules through the intercalation of polar organic molecules. As the Kaol interlayer space expands, the structural disorder decreases and the interlayer bonding force weakens. Kaol breaks down into smaller particles, which increases its surface area and dispersibility. Zhang et al.^[^ ^118^ ^]^ obtained Kac‐modified Kaol using a mixture of Kac and Kaol in a mass ratio of 1:1 and grinding them in a planetary mill at 400 rpm for 1 h. This process doubled the pore volume, increased the average pore size from 15.7 to 23.3 nm, and enhanced the pore size of the capturing capacity of the Kaol‐Kac from 77 to 100 mg g^−1^. DMSO, a strong polar solvent, is the most common guest compound for Kaol intercalation, and the use of DMSO has laid the foundation for further modifications. An exploration of the mechanism of DMSO intercalation indicated that the DMSO molecule forms hydrogen bonds between the sulfonyl oxygen atoms and the Kaol hydroxyl groups, which locks the DMSO above the octahedral sheet pores, while the two methyl groups in the molecule form additional bonds with the oxygen atoms on the tetrahedral surface, leading to the ability of DMSO to arrange itself between the Kaol layers in an orderly manner.^[^ ^119^ ^]^ Further, the adaptive biasing force method was used to calculate the transfer free energy of DMSO molecules between and around Kaol layers in accelerated molecular dynamics simulations. Both the octahedral and tetrahedral surfaces between Kaol layers were observed to exhibit strong DMSO affinity, which helps DMSO in breaking the energy barriers to enter the Kaol interlayers.^[^ ^106^ ^]^ Moreover, the preparation of environment‐friendly Kaol will aid in promoting the application of nanohybrid Kaol as low‐ or zero‐toxicity drug carriers. A stable hydrated Kaol with *d*_(001)_ = 0.84 nm was prepared successfully by heating the transition phase to form hydrazine‐hydrate–intercalated Kaol,^[^ ^120^ ^]^ which can be used as a superior precursor for the preparation of other Kaol intercalates, such as the Kaol‐glycine intercalate.

### 3.3 Grafting Modification

**Figure 6:**
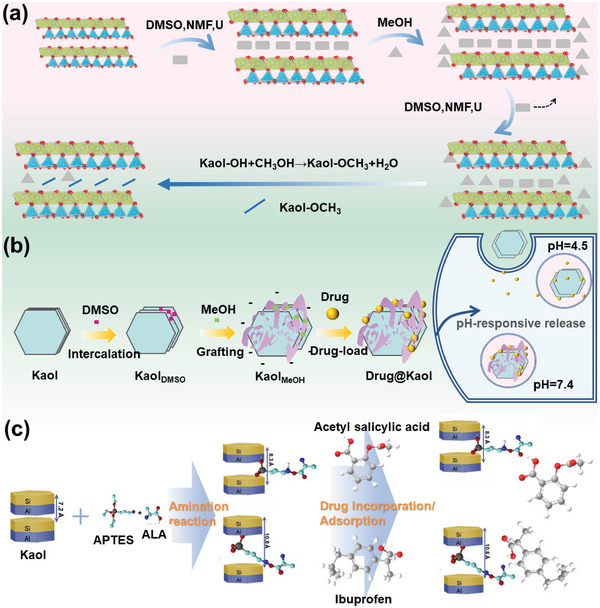
a) Structural evolution models of methoxy grafting of Kaol with three types of pre‐intercalated methoxy‐grafted Kaol derivatives. Reproduced with permission.^[^ ^98^ ^]^ Copyright 2017, Elsevier. b) Synthetic route of Kaol_MeOH_ and Drug@Kaol_MeOH_, and the drug is released at various pH values (pH  =  7.4, 5.5, and 4.5). Reproduced with permission.^[^ ^116^ ^]^ Copyright 2016, Elsevier. c) Synthetic route of APTES‐modified Kaol as adsorbents using Kaol, acetylsalicylic acid, and ibuprofen. Reproduced with permission.^[^ ^122^ ^]^ Copyright 2020, Elsevier.

The process of grafting organic compounds onto activated alumina of Kaol via covalent bonding is called a grafting modification. To date, no organic matter has been directly grafted onto the Kaol interlayer; therefore, grafting modification requires the selection of a suitable Kaol precursor. The preparation process generally involves the preparation of DMSO‐, U‐, and NMF‐modified Kaol precursors^[^ ^121^ ^]^ followed by the preparation of Kaol_MeOH_ through esterification‐like reactions (**Figure** **6**a). Materials prepared from Kaol via grafting have modified physical and chemical properties when compared to the parent Kaol. Moreover, the grafting reaction extends the range of guest substances intercalated with Kaol and can include APTES, ammonium acetate, lactam, MeOH, and other organic substances. Different types and shapes of Kaol can be synthesized using Kaol_MeOH_ as an intermediate and a drug carrier, thereby achieving a sufficient increase in the layer spacing to accommodate drug molecules. The DMSO molecules, for example, widened the substrate spacing of Kaol for MeOH to smoothly enter the interlayer and grafted onto the aluminol surface through Al—O—C bond grafting on the Kaol interlayer. Zhang et al.^[^ ^116^ ^]^ reported that MeOH enabled the interlayer of Kaol to be extended from 0.72 to 0.85 nm, which led to the availability of an additional loading site for the intercalation of the drug. Meanwhile, the increased negative surface charge due to MeOH promoted the loading of the positively charged anticancer drug, DOX (Figure 6b). Moreover. MeOH modification can be used as an intermediate to prepare Kaol‐APTES,^[^ ^123^ ^]^ which has a high affinity and adsorption capacity of Kaol‐APTES with acetylsalicylic acid and ibuprofen: 10.20 mg g^−1^ for Kaol against 210.30 mg g^−1^ for Kaol‐APTES, thus affirming the drug adsorption enhancement by grafting modification (Figure 6c).^[^ ^122^ ^]^

### 3.4 Exfoliation Modification

**Figure 7:**
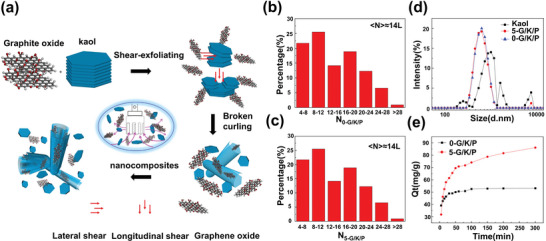
a) Schematic of the exfoliation of Kaol using a high‐shear mixer with graphite oxide as a dispersant. The Kaol average layers of mechanical exfoliation are about b) 14, and mechanical and liquid‐phase exfoliations are less than c) 5. d) Particle‐size distribution and e) methylene blue adsorption by Kaol after mechanical and liquid‐phase exfoliations at different times. Reproduced with permission.^[^ ^128^ ^]^ Copyright 2019, Elsevier.

The close‐packed structure of Kaol creates large particles, is poorly homogeneous, and has limited exposed surfaces, which limits its application.^[^ ^124^ ^]^ Exfoliation modification causes the weak hydrogen bonds between Kaol interlayers to break under an external force, change their structure, and become delamination layers with lower strength and nanoscale size for drug‐delivery applications. Kaol exfoliation methods include mechanical grinding,^[^ ^125^ ^]^ liquid‐phase exfoliation,^[^ ^126^ ^]^ and chemical pre‐intercalation.^[^ ^127^ ^]^ The mechanical grinding technology is relatively mature, and is primarily based on the joint movement of the grinding media and Kaol in an aqueous medium, resulting in shearing, squeezing impact, and abrasive stripping effects. Liquid‐phase exfoliation is a method used for stripping Kaol layers using liquid modifiers, such as DMSO, MeOH, APTES, and DDA. Furthermore, the combination of mechanical and liquid‐phase methods can enhance the exfoliation effects of Kaol (**Figure** **7**a). Mechanical exfoliation reduced the particle size of Kaol from 960 to 610 nm with an average of 14 layers,^[^ ^128^ ^]^ whereas the graphite oxide–assisted mechanical and liquid‐phase exfoliation further reduced the particle size to 530 nm with less than five layers (Figure 7b,d). The increased number of stripping layers amplified the adsorption capacity of Kaol for methylene blue increased from 110 to 250 mg g^−1^ (Figure 7e).^[^ ^128^ ^]^

**Figure 8:**
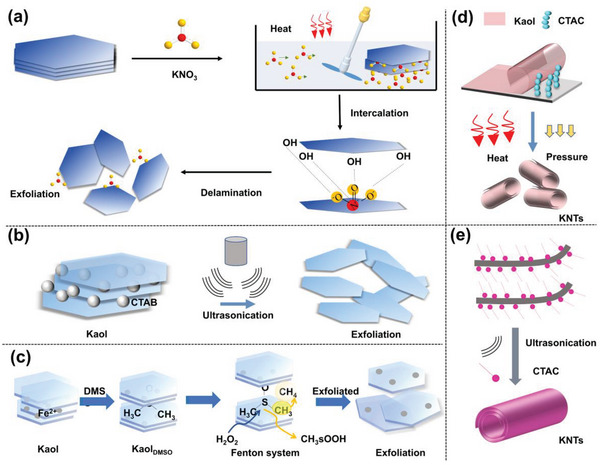
a) Simple exfoliation modification of Kaol. Reproduced with permission.^[^ ^129^ ^]^ Copyright 2019, Elsevier. b) Exfoliated Kaol with thin particles and increased surface area. Reproduced with permission.^[^ ^130^ ^]^ Copyright 2020, American Chemical Society. c) The exfoliation of organic‐free Kaol was performed through an interlayer Fenton reaction. Reproduced with permission.^[^ ^131^ ^]^ Copyright 2019, John Wiley and Sons. d) Transformation of Kaol from a lamellar structure to a tubular structure with a combined effect of heat and pressure. Reproduced with permission.^[^ ^83^ ^]^ Copyright 2022, Elsevier. e) Combined effect of Ultrasonication and cetyltrimethylammonium chloride modifier. Reproduced with permission.^[^ ^85^ ^]^ Copyright 2019, Elsevier.

The mechanical methods, such as heating, pressurization, and ultrasonication, also effectively promoted the extrusion of modifiers into the Kaol interlayer. Exfoliated Kaol nanosheets were successfully stripped using KNO_3_ combined with stirring and heating processes (**Figure** **8**a).^[^ ^129^ ^]^ Tian et al.^[^ ^130^ ^]^ used CTAB in conjunction with ultrasonication to form exfoliated Kaol nanolayers at 60 °C (Figure 8b), which resulted in an enhanced surface area of 104 m^2^ g^−1^ and an increase in EE capacity to 670 mg g^−1^. Moreover, halloysite, which belongs to the Kaol family, is widely used in the biomedical field because of its tubular structure; thus, Kaol applications can be improved by changing its structure from a plate to a tube by exfoliation modification.^[^ ^132^ ^]^ CTAC intercalation resulted in delamination and rolling of Kaol_MeOH_; the Kaol sheets were transformed into nanotubes by employing increased pressure and temperature in a hydrothermal reactor (Figure 8d),^[^ ^83^ ^]^ exhibiting a large aspect ratio, specific surface area, and total pore volume. Exfoliation was demonstrated in the application of encapsulation, and controlled and sustained release of functional molecules.^[^ ^85^ ^]^ The rolling of Kaol resulted from the mutual interaction of CTAC and ultrasound to produce KNTs (Figure 8e), which were successfully used as nanocarriers for 5‐FU with an increasing EE capacity as compared to pure Kaol.^[^ ^86^ ^]^ Moreover, the exfoliation without organic residues was considered due to the need for drug carriers in vivo. H_2_O_2_ entered the Kaol_DMSO_ and forms a Fenton system with Fe^2+^ in situ.^[^ ^131^ ^]^ In the presence of DMSO, hydroxyl radicals in a solution can be quantitatively converted to CH_3_ radicals and methane sulfite intermediates. CH_3_ radicals extract hydrogen from DMSO or methane sulfite, generating large amounts of CH_4_ gas and facilitating Kaol exfoliation (Figure 8c).

## 4 Biosafety Evaluation of Kaol

In vivo toxicology allows the study of complex interactions among nanostructures, tissues, and organs in animal models, providing information on the biodistribution, clearance, immune response, and metabolism. Biomedical applications of Kaol are of increasing interest, and several biological studies are required to confirm its applicability in clinical applications. Several studies have examined the in vitro toxicological properties of Kaol with various modifications and the nanocomposites derived from these modifications.^[^ ^133^, ^134^, ^135^ ^]^ On the one hand, in vitro trials are primarily used to screen and generate more comprehensive toxicological data, providing evidence of biological damages and elucidating the action mechanism responsible for the toxic effects. On the other hand, the results of in vitro trials must be verified using in vivo experiments. Therefore, the biocompatibility of Kaol was assessed using cytocompatibility, histocompatibility, and hemocompatibility tests in combination with in vitro and in vivo tests.

### 4.1 Cytocompatibility of Kaol

Cells are the basic structural and functional units in living organisms; therefore, cytocompatibility assessment of Kaol materials aids in evaluating the effect of Kaol as a drug carrier in body tissues. Notably, Kaol clay nanocarriers can only be cytotoxic to cells at the implantation site because of direct contact after entering the matrix; however, the cellular activity through ion release for leaching toxins is unaffected.^[^ ^136^ ^]^ In a cancer cell model, the cytotoxicity of Kaol was assessed by cultivating the human lung cancer cell line A549 with Kaol for 24 h after methotrexate reduction; the results showed a semi‐inhibitory concentration of 26.6 ± 1.1 µg mL^−1^ for Kaol, indicating that Kaol was less toxic to A549 cells.^[^ ^137^ ^]^ However, according to flow cytometry data, the metabolic activity of A549 cells under the effect of Kaol was relatively low. Moreover, the toxicity of Kaol was determined using several human cancer cell models. The biological responses of human cancer cell models representing various cancers, including lung, colon, gastric, breast, pancreatic, cervical, prostate, esophageal, and differentiated thyroid cancers, were tested using Kaol at a concentration of 200 µg mL^−1^. The results showed almost no biological responses, with the cell viability of the tumor cell lines being more significant at values ≥85%. The highest and lowest cell viabilities were 99.0% and 61.3% for esophageal and lung cancers, respectively. In a non‐cancer cell model, the cytotoxicity of Kaol was evaluated by applying Kaol on rat dermal fibroblast (RDF) cells, and the study showed that Kaol did not have any toxic effect on RDF cells; moreover, it also reduced the toxic effect of graphene oxide, resulting in a significant decrease in the percentage of apoptotic cells.^[^ ^138^ ^]^ Furthermore, in a human lymphocyte cytotoxicity assay, non‐toxicity of Kaol to human lymphocytes was observed at doses ranging from 3.9 to 125 µg mL^−1^. In comparison, cell viability remained greater than 85% at doses ranging from 125 to 500 µg mL^−1^, which proves that Kaol can be characterized as a biocompatibile material for healthy cells.^[^ ^139^ ^]^

**Figure 9:**
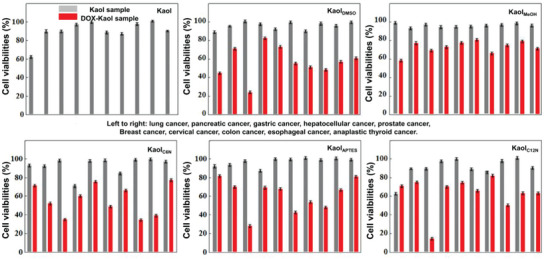
Viabilities of ten model cell cultures when incubated with Kaol at different modifications as assessed using MTT analysis. Reproduced with permission.^[^ ^89^ ^]^ Copyright 2017, Tsinghua University Press.

In an in vitro study on the genotoxicity of Kaol, an in vitro assay of Kaol micronuclei was performed using the human lung cancer cell line A549. The results showed that the A549 cells were subjected to 60% growth inhibition after 6 h incubation with 200 µg mL^−1^ of Kaol; moreover, Kaol exhibited genotoxicity in an in vitro assay system;^[^ ^140^ ^]^ However, when the genotoxicity of Kaol nanomaterials was studied using the DNAs comet assay, after incubation of 100 µg mL^−1^ Kaol with A549 cells for 24 h, more than 50% of the cells suffered only 10% growth inhibition and the DNAs damage was significantly less than that caused by DOX, carbon nanotubes, and graphene.^[^ ^137^ ^]^ Genotoxicity studies were performed using *Caenorhabditis elegans* (*C. elegans*), and no morphological changes, such as rod deformation, vacuolization, or spreading of the macronucleus were observed after Kaol treatment of *C. elegans*. Finally, the induction of oxidative stress in Kaol‐treated cells was monitored based on malondialdehyde concentration measurements and catalase—an antioxidant enzyme—activity. The results showed that 10 mg mL^−1^ of Kaol marginally increased the propylene glycol concentration with hardly any increase in catalase activity.^[^ ^141^ ^]^ In vivo genotoxic effects of Kaol were analyzed using comet and mutation assay systems on gpt delta transgenic mice. The results showed that DNAs loss was not induced by Kaol even at 24 h of exposure time. However, the mutagenicity of gpt and Spi mutations was demonstrated in the gpt delta transgenic mouse system, which may be due to Kaol‐induced DNAs damage.^[^ ^140^ ^]^ *C. elegans* are often used as a viable model for verifying the acute or long‐term toxicity of various compounds, including nanomaterials;^[^ ^141^ ^]^ moreover, protozoa were observed to exhibit positive chemotaxis to Kaol in a protozoan chemotaxis assay; subsequently, the acute toxicity of Kaol after ingestion by the protozoa was evaluated. The results showed that Kaol was not toxic at lower concentrations and even stimulated cell growth to a certain extent. Kaol modification treatments have paved the way for its application in anticancer drug transport, and anti‐inflammatory and antibacterial applications. **Figure** **9** shows the viabilities of ten model cell cultures incubated with modified Kaol, which are assessed via 3‐(4,5‐dimethylthiazol‐2‐yl)‐2,5‐diphenyl‐2H‐tetrazolium bromide (MTT) analysis. Among these, DMSO‐modified Kaol is the most widely used because it is often used as the first step before other modifications. The cytotoxic effect of DMSO‐intercalated Kaol on human lymphocytes was determined using the MTS Braford test, which demonstrated that the cytocompatibility of Kaol was enhanced by DMSO modification.^[^ ^139^ ^]^

### 4.2 Histocompatibility of Kaol

Nanomaterials can enter the human body through implantation, inhalation, dermal uptake, and intravenous injection. Owing to their small size, they can migrate widely in the body, even through the blood–brain barrier, and may cause toxicity in the kidney, liver, spleen, lung, gastrointestinal tract, skin, and other target organs after metabolism by various tissues. The results of a therapeutic study on the application of the Kaol‐based nanocomposite FePt@Kaol‐DOX in 6 weeks‐old male non‐obese diabetic/severe combined immunodeficient interleukin‐2 receptor gamma null (NSG) mice indicated that a tail vein injection of 10 mg kg^−1^ FePt@Kaol‐DOX combined with a magnetic field induced its accumulation in tumor tissues. Hematoxylin and eosin staining results showed that significant damage was observed only in the tumors and tissues, including the heart, liver, spleen, lungs, and kidneys. No significant pathological changes were observed in tissue sections of the organs.^[^ ^91^ ^]^

### 4.3 Blood Compatibility of Kaol

ECs covering the lumen of blood vessels are the first points of contact for Kaol before it reaches its target through the blood pathway. Kaol was incubated with human umbilical vein ECs (HUVECs), which can be considered as a classical EC model, and mouse macrophages (RAW 264.7) at 37 °C, and the cell viability was assessed using alamarBlue reagent. For 100 µg mL^−1^ of Kaol dose, 40% of HUVECs survived for more than 24 h. At the same time, cellular toxicity to RAW 264.7 was negligible. Notably, the effect of Kaol on HUVEC viability was still lower than that of the U.S. Food and Drug Administration–approved and commercially marketed topical hemostat agent called WoundStat.^[^ ^136^ ^]^ The results of an erythrocyte hemolysis assay showed that the particle size of Kaol influenced the hemolysis rate, with particle sizes ranging from 0.27 to 1.11 µm having a lower hemolysis ratio than those in the range of 1.68 to 3.44 µm.^[^ ^52^ ^]^ Kaol particles with a size of 0.27 µm were well dispersed and maintained the typical biconcave shape after 5 h of incubation with whole blood. Kaol exhibits high hemocompatibility in addition to inducing platelet adhesion, activation, and aggregation in the blood,^[^ ^139^, ^142^ ^]^ whereas the percentage of hemolysis of DMSO‐modified Kaol was less than 5%, proving its equally high hemocompatibility.

Numerous studies have shown that the toxic effects of nanomaterials arise from adverse absorption, distribution, metabolism, and excretion (ADME) processes in living organisms. Although the biocompatibility of Kaol as a biomedical material has been demonstrated in cell, tissue, and blood compatibility assessments, this biocompatibility is closely related to the cell type, duration of action, and concentration of action. Therefore, ADME, pharmacodynamic, and biological persistence studies are required to understand the preclinical Kaol biological data and facilitate further clinical trials.

## 5 Therapeutic Delivery of Kaol

Owing to its natural nanolayer structure, abundant active hydroxyl groups, variable charge, hydrophilic properties, and other physicochemical properties, Kaol is a suitable nanocarrier for the loading and controlled release of various guest drugs (e.g., antibiotics, anticancer drugs, antioxidants, and anti‐inflammatory drugs). Moreover, metal or metal‐oxide NPs with specific functions can be assembled on the surface or between layers of Kaol, combining the properties of Kaol and the optical, magnetic, catalytic, and adsorption properties of the NPs to exert synergistic effects in Kaol‐based delivery for therapeutic applications. Recent research has focused on the development of multifunctional DDSs based on the structural and surface properties of Kaol to modulate the loading guest to improve efficacy and achieve additional functionality from a therapeutic point of view, with major application areas including antibacterial, anti‐inflammatory, and antitumor therapy.

### 5.1 Kaol‐Based Carrier for Antibacterial Treatment

**Figure 10:**
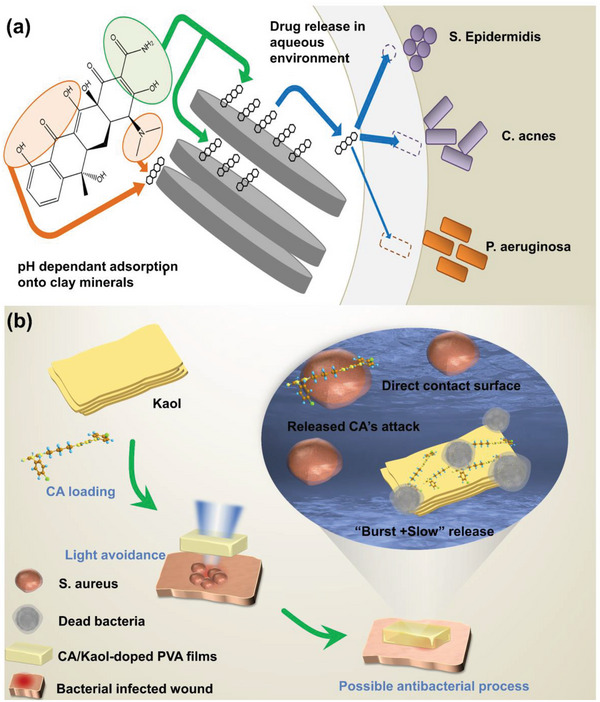
a) Antibiotic‐loaded Kaol exhibited drug‐release and antibacterial effects. Reproduced with permission.^[^ ^143^ ^]^ Copyright 2019, Elsevier. b) Antibacterial advantage and possible antibacterial mechanism of CA/Kaol‐doped PVA films. Reproduced with permission.^[^ ^59^ ^]^ Copyright 2019, Elsevier.

Bacteria can cause delayed wound healing or wound deterioration forming bacterial wound infections. This is due to disruption of the outer epidermal barrier of the wound, which, combined with the denaturation of proteins and lipids, provides a fertile environment for bacterial growth, triggering infection of the immune system and leading to inflammation and delayed healing. Although there was apparent self‐healing in most cases of infected wounds, untreated or improperly treated severe wounds may persist and become life‐threatening. Therefore, new topical delivery systems must be urgently developed to suppress or eradicate pathogenic bacteria while promoting wound healing. The use of Kaol as a carrier loaded with antibiotics, antimicrobial agents,^[^ ^143^ ^]^ or NPs^[^ ^144^ ^]^ is effective in treating bacterial wound infections due to its natural 2D layer structure, abundance low toxicity, and surface polarization. The potential of Kaol as a substance for antibiotic administration to wounds was shown by the drug release of tetracycline (TC) and doxycycline (DC) on the Kaol surface.^[^ ^143^ ^]^ TC and DC had a positive charge at pH 2 or lower and were more likely to adsorb onto the negatively charged Kaol surface. Also, the antibiotic‐loaded composite showed antibacterial activity against *Staphylococcus epidermidis*, *Clostridium acnes*, and *Pseudomonas aeruginosa*. The above results demonstrate that Kaol is a suitable antibiotic delivery material that releases TC and DC effectively and had significant antibacterial effects proportional to the antibiotic load (**Figure** **10**a). Chlorhexidine acetate (CA) is a superior antimicrobial agent whose cations can bind to negatively charged bacterial cell membranes to achieve antimicrobial effects; however, it cannot be used directly as an antimicrobial product and must be loaded onto carriers to achieve controlled release and better dispersibility. It was found that Kaol achieved a combination of burst release and sustained slow release of CA, with rapid sterilization in the burst phase and long‐lasting sterilization in the sustained release phase.^[^ ^59^ ^]^ Furthermore, the CA‐loaded Kaol encounters the bacteria through the triple adsorption phenomena of mineral, electrostatic, and hydrophobic adsorptions. For *Staphylococcus aureus* (*S. aureus*), which is prone to inducing wound infections, the minimum inhibitory concentration was 11.72 mg L^−1^, and an antibacterial rate of 98.37% was achieved by contact inactivation and CA‐release–based sterilization. A subsequent study on the CA/Kaol for the preparation of antimicrobial wound dressings showed that Kaol increased the opacity of the dressing and improved the light‐avoidance effect; moreover, the inhibition zones of CA/Kaol in the dressing with up to 30% doping were 15.393 mm for *S. aureus*, confirming that CA/Kaol is an antimicrobial material with superior antimicrobial activity and processability (Figure 10b). Moreover, NPs (e.g., Ag,^[^ ^145^ ^]^ Au,^[^ ^146^ ^]^ and ZnO^[^ ^53^ ^]^) have been used for bactericidal effects against multiresistant and biofilm‐forming bacteria, but the use of NPs for antimicrobial purposes suffer from problems such as aggregation, crystal growth, or surface morphology changes, which ultimately reduce their antimicrobial efficiency and limit the industrial application. Kaol not only can effectively overcome these problems by immobilization of NPs but Kaol also a perfect nanoparticle delivery system that can control the leaching of metal NPs to a concentration below the limit of toxicity to human cells and maintain the bactericidal activity for a longer period.^[^ ^147^ ^]^ Kaol nanocomplexes loaded with Ag NPs (Ag/Kaol/Chit) showed antimicrobial activity and improved wound healing ability by reducing inflammation and promoting cell proliferation. Kaol lamellar nanospace also was used to load Fe_2_O_3_ NPs with antimicrobial activity, providing domain‐limited space for preventing the aggregation of high‐surface‐energy NPs during treatment, thus enhancing the antimicrobial activity of nanostructured metal oxides.^[^ ^148^ ^]^

### 5.2 Kaol‐Based Carrier for Anti‐Inflammatory Treatment

**Figure 11:**
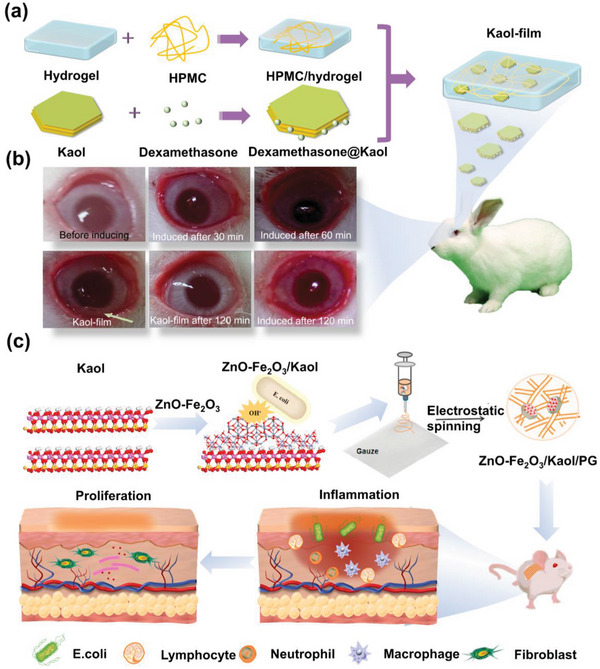
a) Model diagram of conjunctival anti‐inflammatory study in the eyes of rabbits treated by Kaol‐film. b) After 120 min the disappearance of redness and tearing of the eye treated by Kaol‐film has been observed in contrast to continued conjunctival inflammation without Kaol‐film. a,b) eproduced with permission.^[^ ^56^ ^]^ Copyright 2018, Taylor and Francis. c) ZnO NPs were anchored on the surface of a Fe_2_O_3_/Kaol composite and mediated the inhibition of the release of pro‐inflammatory cytokines. Reproduced with permission.^[^ ^53^ ^]^ Copyright 2021, Elsevier.

Inflammation is a pathological process that occurs when biological tissues are stimulated by trauma, infection, or irritants. Kaol, a natural mineral, has a palliative effect on inflammation, especially ulcerative colitis, and acts as a drug carrier to play a synergistic role in the treatment of inflammation.^[^ ^149^ ^]^ In vitro delivery therapy of Kaol, it can protect mucosal membranes by adsorbing poisons, germs, and even viruses. Dexamethasone acetate is used as an eye medication^[^ ^150^ ^]^ which is used topically to treat proliferative vitreoretinal lesions by lessening the ocular inflammation and disruption of the blood‐ocular barrier.^[^ ^151^ ^]^ The ocular administration of dexamethasone was enhanced by using Kaol together with dexamethasone polymer implants (Kaol‐film).^[^ ^56^ ^]^ Cation exchange and hydrogen bonding in Kaol allowed the adsorption of the drug onto the surface of the Kaol, thus prolonging the drug penetration (**Figure** **11**a). The presence of Kaol also prolonged the ocular penetration of the drug by more than 6 h, and the anti‐inflammatory efficacy of the Kaol‐film was confirmed via an anti‐inflammatory test of rabbit eye conjunctiva (Figure 11b). Furthermore, Kaol is equally capable of assisting wound inflammation healing in inflammation treatment in vitro (Figure 11c). ZnO‐loaded Kaol (ZnO/Kaol) released Zn^2+^ and reduced lipopolysaccharide‐induced production of the pro‐inflammatory mediators NO and tumor necrosis factor‐*α* in TNF‐*α* in RAW264.7 macrophages. Further histological analysis of wound healing processes, it was evident that ZnO/Kaol‐treated wounds showed almost complete integration of the epithelial tissue after 14 days, with only a few inflammatory cells remaining, except in the case of the gauze group. The undecorated gauze‐treated wounds contained a large number of inflammatory cells that maintained the inflammatory response, confirming the anti‐inflammatory effect of ZnO/Kaol.^[^ ^53^ ^]^ The advantages of Kaol as a topical anti‐inflammatory, besides being a carrier to carry anti‐inflammatory molecules, are the high thermal retardation capability, which allows the accumulation and collection of excess biochemical fluids in the swollen tissues, inhibiting edema at the site of inflammation and thus reducing pain and congestion. The high insulating capacity of Kaol allowed vasodilation and increased blood flow from the skin to the site of infection, leading to heat dissipation. Still, in the case of inflammation triggered by bacterial infection, this affects the increase in coagulability and pain intensity and is a deficiency of Kaol in the treatment of wound inflammation.

### 5.3 Kaol‐Based Carrier for Anticancer Treatment

Owing to its deadly nature, cancer requires urgent breakthroughs in terms of early diagnosis and improved treatment. A primary cancer treatment method involves the use of anticancer drugs. Kaol can be a possible solution to overcome existing problems owing to its tunable physicochemical, morphological, and structural properties. Its particle size and high specific surface area allow for the effective loading of drugs and the ability to sustain the controlled release and delivery of anticancer drugs to tumor locations. Moreover, Kaol promotes cell–cell and extracellular matrix adhesion between cancer cells and prevents cancer cell metastasis.^[^ ^152^ ^]^ Therefore, Kaol provides a high‐performance theranostic platform for the efficient theranostic treatment of tumors and paves the way for integrating various functional moieties to achieve multifunctional therapy in tumor treatments.

**Figure 12:**
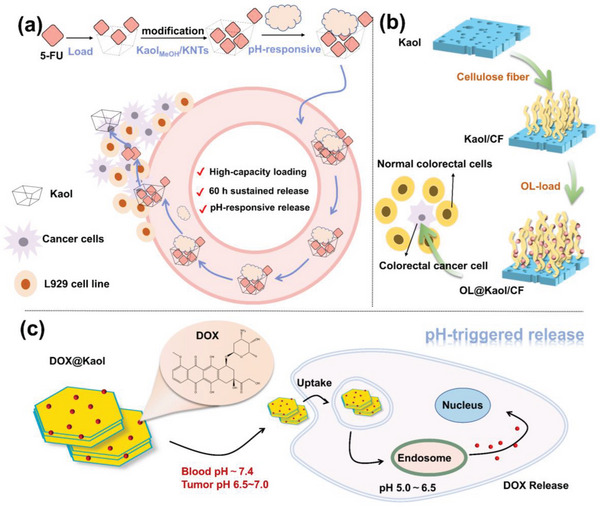
a) Role of Kaol as drug carrier in 5‐FU loading. Reproduced with permission.^[^ ^86^ ^]^ Copyright 2019, Elsevier. b) Schematic illustration of the treatment of colorectal cancer of Kaol/CF after OL‐loaded. Reproduced with permission.^[^ ^130^ ^]^ Copyright 2020, American Chemical Society). c) Schematic illustration of the pH‐responsive targeted chemotherapy of Kaol after DOX loading. Reproduced with permission.^[^ ^81^ ^]^ Copyright 2019, Springer).

The drug 5‐FU is commonly used in cancer chemotherapy^[^ ^153^ ^]^ (including breast, gastric, pancreatic, colon, and rectal cancers). It interferes with nucleoside metabolism and acts as a pyrimidine analog, replacing uracil or thymine and incorporating it into the ribonucleic acid and DNAs, leading to cytotoxicity and cell death.^[^ ^154^ ^]^ However, its half‐life is only 10–30 min; consequently, repeated dosing is required. Loading the drug onto Kaol nanocarriers prevented early biodegradation of 5‐FU and improved the effectiveness of the drug (**Figure** **12**a). Tan et al.^[^ ^58^ ^]^ concluded that 5‐FU can be intercalated in an amorphous form into Kaol layers, thus avoiding thermal decomposition and outward diffusion of 5‐FU, which allows the prepared Kaol nanocarriers to load and control the selective release of 5‐FU. M. R. Abukhadra et al.^[^ ^86^ ^]^ conducted in vitro release and in vivo toxicity experiments on KNTs loaded with 5‐FU (5‐FU@KNTs); the results showed that the 5‐FU released from the KNTs was sustained for 60 h (Figure 12a). The cytotoxicity of 5‐FU@KNTs was also evaluated using the MTT assay and L929 cell line, which confirmed an average cell viability of 98.6%; thus, KNTs can be used as efficient drug carriers for 5‐FU.

Oxaliplatin (OL) is a chemotherapeutic agent used for the treatment of colorectal cancer that acts on DNAs by producing hydrated derivatives for the formation of cross‐links between chains, thereby inhibiting DNAs synthesis and achieving cytotoxic effects and antitumor activity. However, its drawbacks, such as low solubility, narrow therapeutic window, and poor distribution pattern, lead to similar toxic effects on both cancer and normal cells. Kaol, as a standard drug carrier, can be used as an advanced means to control drug release and ensure drug safety. Tian et al.^[^ ^130^ ^]^ confirmed that a composite of Kaol and cellulose fiber (Kaol/CF) can be used as a drug carrier by studying the effect of monolayer‐modified Kaol/CF on OL drug‐loading performance, control mechanism, in vitro drug release, and cytotoxicity (Figure 12b). The composite was observed to be an effective carrier for the induction of safe drug molecules and the achievement of therapeutic efficacy. Moreover, OL‐loaded Kaol/CF was beneficial for cancer cell therapy, with cell viability of 77% for the free form of the colorectal cancer cell and 31.4% for cells treated using OL@Kaol/CF.

DOX is a broad‐spectrum antitumor drug that induces cytotoxicity by inducing DNAs double‐strand breaks^[^ ^155^ ^]^ in a variety of cancer‐related diseases, such as malignant lymphoma and breast, bladder, thyroid, testicular, gastric, and liver cancers. However, DOX has several side effects, including rapid clearance, poor tumor selectivity, and irreversible cardiotoxicity. To improve the effectiveness and safety of DOX, considering that DOX particle size is between 0.28 and 0.84 nm, researchers extensively used modified Kaol for DOX delivery. The schematic diagram of its delivery mechanism was shown in Figure 12c.^[^ ^156^, ^157^ ^]^ An in vitro study on the binding and release mechanisms of DOX using pure Kaol revealed that DOX preferentially adsorbs on the outer surface of Kaol and then on the inner surface of Al—OH rather than on Si—O. The protonation of negatively charged Kaol in weakly basic solutions resulted in the pH‐responsive release of the Kaol drug carrier,^[^ ^158^ ^]^ which showed that the positively charged DOX and negatively charged Kaol are strongly electrostatic, exhibiting a low release rate for DOX under simulated normal physiological conditions (pH = 7.4). In contrast, the simulated tumor environment (pH = 5.0) caused weakened electrostatic interactions between the Kaol interlayers and resulted in a faster DOX release rate. This pH‐responsive controlled‐release behavior may facilitate targeted drug delivery to cancer tissues while reducing drug side effects.^[^ ^89^ ^]^ Kaol and its modification products can be internalized into the cytoplasm by endocytosis in a time‐dependent manner, and DOX‐loaded Kaol nanocarriers can further enter the lysosomes without passing through the nuclear membrane.

**Figure 13:**
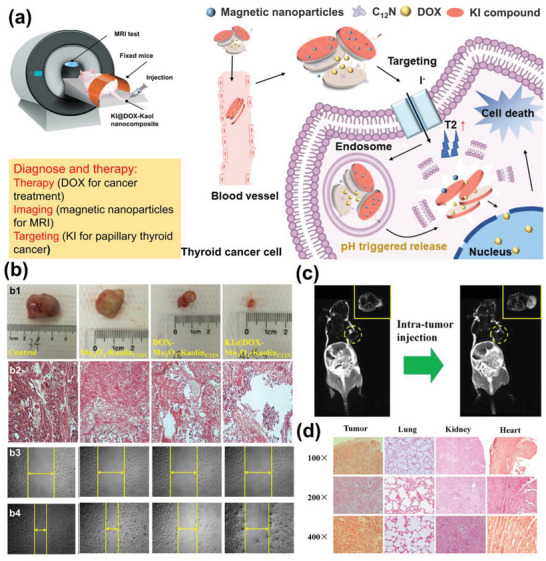
a) Schematic diagram of KI@DOX‐Mn_3_O_4_‐Kaol_C12N._ b) Digital photos (b1) and tumor‐volume change and microscopic images (b2) of tumor sections of tumor‐bearing nude mice after the implantation and antimetastasis assay results (b3,b4) of control, Mn_3_O_4_‐Kaol_C12N_, DOX‐Mn_3_O_4_‐Kaol_C12N_, and KI@DOX‐Mn_3_O_4_‐Kaol_C12N_. c) In vivo imaging before and after (0 and 5 min, respectively) intra‐tumor administration of KI@DOX‐Mn_3_O_4_‐Kaol_C12N_ (top‐right corner shows magnified images of corresponding circled area). d) Microscopic sectional images and biodistribution of KI@DOX‐Mn_3_O_4_‐Kaol_C12N_ in tumor and main tissues. Reproduced with permission.^[^ ^81^ ^]^ Copyright 2019, Springer.

Chemotherapy is the most widely used cancer treatment method. Kaol nanocarriers have been successfully loaded with chemotherapeutics, such as DOX, 5‐FU, and OL, showing promise as drug delivery vehicles and direct antitumor systems. However, a major limitation of current cancer therapies is the poor delivery of anticancer therapeutics to specific cancer‐targeted tissues/cells, which must be appropriately designed to maximize efficacy. Targeted nanocarriers can transport chemotherapeutic drugs to specific tumor‐targeted tissues/cells while avoiding indiscriminate attacks on healthy tissues/cells. A multifunctional KI@DOX‐Mn_3_O_4_‐Kaol_C12N_ nanocarrier was constructed to achieve magnetic resonance imaging and chemotherapeutic drug delivery in a single method (**Figure** **13**a).^[^ ^81^ ^]^ These nanocomposites mainly used DDA‐modified Kaol (Kaol_C12N_) as an intermediate, loaded with manganese tetroxide magnetic NPs as a tumor marker for T1 MRI‐based detection (Figure 13c), and were coated with potassium iodide for targeted function via iodine uptake by the thyroid gland. The nanocomposites significantly reduced the cell viability of papillary thyroid cancer and reduced the tumor size by 75% (Figure 13b1,b2). An antimetastasis assay demonstrated that the complex almost completely inhibited papillary thyroid cancer cells (Figure 13b3,b4). The final pathological examination measured Al accumulation levels in the tumors, lungs, kidneys, and hearts of mice; KI@DOX‐Mn_3_O_4_‐Kaol_C12N_ accumulation was only found in tumors after 24 h, indicating that these multifunctional nanocomposites were used for postoperative residual therapy and local metastasis observation of thyroid cancer by achieving both dynamic monitoring and targeted therapy through synergistic effects (Figure 13d).

**Figure 14:**
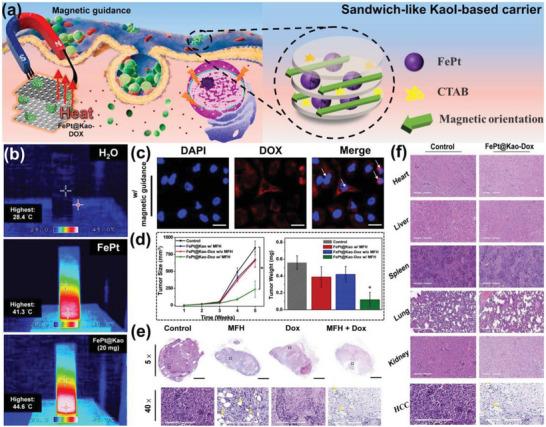
a) Schematic diagram of contact between FePt@Kaol and HCC. b) FePt NPs excite surface free electrons by magnetic resonance in the presence of Kaol to release more energy in the form of thermal energy. c) DOX molecules in the FePt@Kaol‐DOX entered the nucleus after magnetic guidance by FePt. d) Significant reduction in tumor size and weight with dual therapy. e) Control, MFH treatment only, DOX treatment only, and MFH + DOX therapy groups all showed significant damage to the excised tumors. f) Microscopic sectional images and biodistribution of KI@DOX‐Mn_3_O_4_‐Kaol_C12N_ in tumor and main tissues. Reproduced with permission.^[^ ^91^ ^]^ Copyright 2020, American Chemical Society.

FePt NPs can be used to track liver tumor cells because of the easy accumulation of iron in the liver. At the same time, NPs have a T2 shortening effect in MRI, which can stimulate surface free electrons through magnetic resonance to release energy in the form of heat to kill cancer cells. FePt@Kaol‐DOX was obtained by adding DOX to FePt@Kaol nanocomposites in a single method (**Figure** **14**a).^[^ ^91^ ^]^ FePt NPs and DOX enabled the selective accumulation of FePt@Kaol‐DOX in tumor tissues and killed tumor cells via magnetic fluid hyperthermia (MFH) and the pharmacotherapeutic effect of DOX. This Kaol‐based multifunctional DDS, in which Kaol was present not only as a carrier for the adsorbed drug but also as a structure to limit the magnetic vector of FePt, making it more susceptible to external magnetic fields and leading to a more substantial MFH effect (Figure 14b). Thus, magnetic guide material can help FePt@Kao‐DOX movement. While analyzing its accumulation in HepG2 cell line of hepatocellular carcinoma (HCC), more DOX molecules entered the nucleus after magnetic induction, indicating that magnetic induction intensity can be used as a method for specific drug collection in specific tissues or as a method for targeting specific sites (Figure 14c). HCC was selected for in vitro cellular activity and toxicity analysis, and NSG mice were chosen as the experimental model to analyze the biocompatibility and therapeutic effects of FePt@Kaol‐DOX. Tumor reduction was confirmed by a nearly sevenfold reduction in tumor size and a significant decrease in weight (size reduced from 900 to 125 mm^3^ and weight decreased from 0.55 to 0.18 g after the intravenous administration of FePt@Kaol‐DOX) (Figure 14d). Moreover, the results showed that although FePt@Kaol‐DOX was less toxic to HCC, it caused effective damage to HCC cells after magnetic guidance (Figure 14e), demonstrating the targeting of FePt@Kaol‐DOX based on the magnetic effect of FePt NPs (Figure 14f). These results suggest that Kaol can be used as a dual‐efficacy platform for MFH and DOX chemotherapy and effective targeted therapy in HCC.

## 6 Therapeutic Avenues of Kaol

The topical administration of Kaol has been applied in antibacterial and anti‐inflammatory treatment. The intravenous injection is currently the main process of transportation of Kaol‐based carriers for anticancer treatment, which enables rapid delivery and distribution of these materials throughout the vascular system. Still, this mode of delivery carries a toxicity risk due to the potential for thrombosis and the accumulation of aluminum and silica ions.

**Figure 15:**
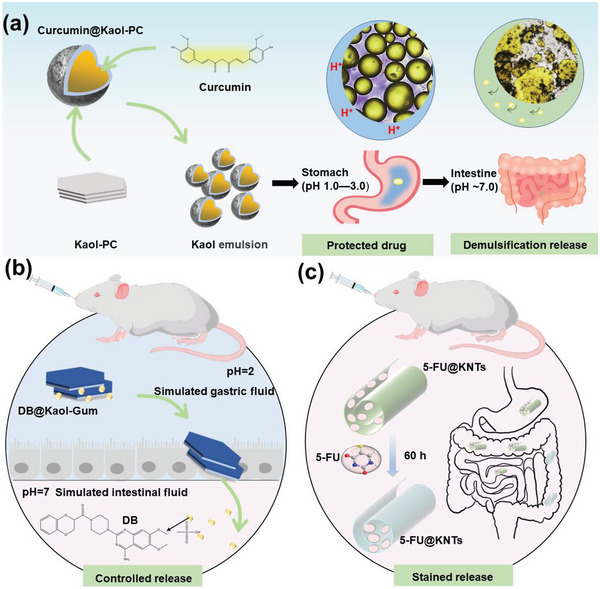
a) Shell structure of Kaol demonstrated the possibility of the manipulation of controlled curcumin release from intestine. Reproduced with permission.^[^ ^90^ ^]^ Copyright 2019, Elsevier. b) Kaol was used to deliver gastrointestinal drugs to the intestine to avoid premature release in the stomach. Reproduced with permission.^[^ ^161^ ^]^ Copyright 2020, Elsevier. c) KNTs have led to attempts at the encapsulation of 5‐FU for sustained release. Reproduced with permission.^[^ ^86^ ^]^ Copyright 2019, Elsevier).

Oral drug delivery is Kaol nanocarriers’ most promising therapeutic route. Kaol can improve drug delivery by increasing drug solubility and bioavailability, controlling and sustaining drug release, and protecting drugs from harsh environments such as stomach acids. Finally, they protect the molecules from rapid degradation, which is an existing problem in drug delivery. Curcumin is a natural bioactive substance with strong antioxidant properties that enable its application in anticancer, anti‐inflammatory, neuroprotective, and anti‐hypertensive treatments.^[^ ^159^ ^]^ However, its low solubility, poor oral bioavailability, and rapid breakdown in alkaline solutions limit its bioavailability in the gastrointestinal tract.^[^ ^160^ ^]^ To address the difficulties in the application of curcumin, Kaol‐PC was used as an emulsifier to stabilize the Pickering emulsion, which resulted in an increase in the bioavailability of curcumin from 16.0% to 80.8%. The Kaol emulsion achieved 81.1% curcumin encapsulation with morphological retention in simulated gastric fluid, confirming the storage stability of curcumin during gastric digestion, while simultaneously being susceptible to lipase in a simulated intestinal fluid, allowing the decomposition of emulsion droplets with higher release efficiency (100%, 150 min) (**Figure** **15**a). Doxazosin mesylate (DB) is used to treat benign plasma augmentation and hypertension. However, its oral administration leads to sudden hypotension after reaching maximum plasma concentration. To avoid a rapid increase in plasma concentration after oral administration of DB and to reduce the frequency of administration, Kaol was chosen as a controlled‐release therapeutic system to improve the therapeutic efficiency of the drug. Silva et al.^[^ ^161^ ^]^ synthesized a Kaol–cashew gum (Gum) nanocomposite using Kaol and Gum, revealing that hydrogen bonding in Kaol promotes DB penetration in the Kaol–Gum nanocomposite. Kaol–Gum protected DB (DB@Kaol–Gum) from acidic conditions, which showed the ability of nanocomplexes to control release, allowing for rapid release under simulated intestinal fluid conditions and slow or no release under gastric fluid conditions. These results show that Kaol–Gum is an effective carrier material for the effective penetration and release of DB (Figure 15b). Research has shown that the lamellar structure can control the release, whereas the tubular structure of Kaol can achieve sustained release to extend the circulation time of 5‐FU (5‐FU@KNTs), demonstrating a controlled‐release that lasted for 60 h at release rates of 74.3% and 91% in the intestinal and colonic fluids, respectively (Figure 15c). These results suggest that Kaol can be used to develop oral delivery.^[^ ^90^ ^]^ Most studies reported to date have emphasized the use of intravenous injection as the primary route, and evaluation of Kaol‐based nanocarriers by oral, intra‐tumor delivery, and dermal routes is very scarce. Understanding and better exploring all these therapeutic avenues in preclinical assays will help develop Kaol‐based nanocarriers with high stability and efficacy in treating most diseases, especially cancer.

## 7 Challenges and Future Prospects

The use of Kaol for DDSs and as a scaffold for carrying NPs and drugs to treat various diseases is at the forefront of its biomedical applications. In this review, up‐to‐date literature concerning the modified methods and biocompatibility of Kaol, especially the material‐physiological environmental relationship between Kaol‐based NPs, drug delivery, and disease therapy is presented. Current studies have shown that it can be used as a nanocarrier to prolong drug delivery time and achieve pH‐stimulated responsive release, which can specifically release drugs in the tumor microenvironment and avoid drug efflux, allowing transporter proteins to recognize anticancer drugs. Targeting ligands, drugs, imaging probes, and proteins can be loaded onto Kaol carriers, providing a multifunctional therapeutic platform for cancer treatment, increasing drug bioavailability, and achieving synergistic effects.

Kaol is the most widely available source of silicate minerals with good biocompatibility. However, the properties of Kaol in terms of geological origin, growth mechanism, and formation process need to be focused on because their composition and properties can vary greatly depending on their natural origins. Kaol crystallinity is highly correlated with geological factors. It is generally believed that Kaol resources are classified as weathering‐, hydrothermal alteration‐, and sedimentary‐type, among which sedimentary‐ and weathering‐type Kaol mostly belong to disordered Kaol. The crystallinity will affect the intercalation modification effect of Kaol, such as highly disordered Kaol is difficult to carry out the intercalation reaction of amide. Moreover, the higher the crystallinity, the higher the surface charge value. This facilitates the adsorption of drug molecules on it to improve the drug loading rate. Furthermore, Kaol of different genesis contains different impurities, such as quartz which can cause the disintegration and death of alveolar macrophages. Using Kaol as a nanocarrier requires mineral pretreatment to separate high‐purity Kaol for biomedical therapeutic needs. Overall, the suitable mineral origin may facilitate the preparation of carrier materials. The next attention is the physical and chemical properties of Kaol, especially, the size of Kaol nanocarriers. The size currently used for animal cancer delivery therapy is 400–750 nm due to the following factors: the first is that intercalation modification is required to expand the layer spacing to fully utilize the 2D lamellar space of Kaol. The elastic deformation generated by the organic molecules penetrating from one end of the nanoparticle will be transmitted to the whole particle faster, thus blocking the intercalation effect. In addition, the interlayer force between nanoscale Kaol layers is also very strong, which is also not conducive to the intercalation reaction; therefore, the size of Kaol carriers favorable for modification cannot be too small. Finally, a carrier should carry functional molecules, causing an increased size. The premise of the guaranteed intercalation effect will lose particle size and lead to embolism in circulation, which will highly limit its biological application in vivo. Therefore, the size threshold of Kaol should be concerned, and the minimum nanosize of Kaol carriers should be determined from the crystal structure and crystal chemistry of Kaol itself under the condition that the intercalation effect and structural properties are not destroyed.

## Conflict of Interest

The authors declare no conflict of interest.

## Author Contributions

H.Y. developed the concept for the review. Q.W. wrote initial drafts of the manuscript. J.L. helped with the manuscript writing. H.Y. revised and wrote the final version of the manuscript. All authors have given approval to the final version of the manuscript.
